# Curcumin and Resveratrol vs. Ferrocene-Modified Polyphenols: Role in Enhancing Protective Properties in Human Keratinocytes

**DOI:** 10.3390/pharmaceutics17121511

**Published:** 2025-11-22

**Authors:** Marina Miletić, Veronika Kovač, Lidija Barišić, Alen Supičić, Bruno Doskočil, Irena Landeka Jurčević, Jelka Pleadin, Branimir Šimić, Ivana Kmetič, Teuta Murati

**Affiliations:** 1 Laboratory for Toxicology, University of Zagreb Faculty of Food Technology and Biotechnology, Pierotti St. 6, 10000 Zagreb, Croatia; mmiletic@pbf.hr (M.M.); alen.supicic@gmail.com (A.S.); bruno.doskocil@pliva.com (B.D.); branimir.simic2@gmail.com (B.Š.); tmurati@pbf.hr (T.M.); 2 Laboratory for Organic Chemistry, University of Zagreb Faculty of Food Technology and Biotechnology, Pierotti St. 6, 10000 Zagreb, Croatia; vkovac@pbf.hr (V.K.); lidija.barisic@pbf.unizg.hr (L.B.); 3 PLIVA Hrvatska d.o.o., Prilaz Baruna Filipovića 25, 10000 Zagreb, Croatia; 4 Laboratory for Food Chemistry and Biochemistry, University of Zagreb Faculty of Food Technology and Biotechnology, Pierotti St. 6, 10000 Zagreb, Croatia; ilandeka@pbf.hr; 5 Croatian Veterinary Institute, Savska St. 143, 10000 Zagreb, Croatia; pleadin@veinst.hr

**Keywords:** curcumin, resveratrol, ferrocene, structural modification, keratinocytes, skin protection, intracellular effects

## Abstract

**Background/Objectives**: Resveratrol (RSV) and curcumin (CRC) have antioxidant, anti-inflammatory, photoprotective, and cell-repairing properties. We selected them to investigate the potential role involved in human keratinocyte protection. Additionally, we tried to overcome the limitations of their application due to poor pharmacokinetics by introducing ferrocene into their structure. **Methods**: The multiple cellular endpoints—viability (determined by MTT and Trypan Blue method), ROS (reactive oxygen species) formation (evaluated by fluorescence intensity measurement), apoptosis and autophagy (assessed using flow cytometry) of *trans*-3,5,4′-tri(4-ferrocenylbutanoyloxy)-stilbene (RF) and (*E*)-5-(4-hydroxy-3-methoxyphenyl)-1-ferrocenylpent-4-ene-1,3-dione (CF), as well as of RSV and CRC, were evaluated in the HaCaT cell line. **Results**: RF and CF showed significantly lower antiproliferative activity, and cell survival was markedly pronounced compared to RSV or CRC-treated cells. CRC exerted the highest cytotoxicity, and cell viability was almost completely impaired at >50 µM. All compounds showed a beneficial effect on the reduction of ROS induced by *t*BHP (*tert*-butyl hydroperoxide), while in UV (ultraviolet) experiments, results were inconclusive (variable depending on dose). CRC and CF had the most prominent antioxidant capacity. Cytofluorimetry showed that CRC has a diverse range of targets in HaCaT cells, including cell proliferation arrest, apoptosis, and autophagy induction. RF at the lowest dose (5 µM) slightly induced autophagy, while treatment with CF even led to a decrease in the autophagy induction ratio. **Conclusions**: Based on the results, introducing ferrocene into natural compounds is an appropriate approach to protect skin cells, considering the low cytotoxicity of ferrocene-modified polyphenols and their retained antioxidant ability. However, caution should be exercised with CRC at ≥20 µM as it significantly impairs cell viability and induces cell death.

## 1. Introduction

Among the great variety of dietary polyphenols (plant nutraceuticals), some constitute a promising approach to remedying many skin conditions [1]. Resveratrol (RSV; Table 1) and curcumin (CRC; Table 1) are representatives of polyphenols with well-documented favorable effects on human health. CRC is effective against chronic inflammation in psoriasis, atopic dermatitis or eczema, while RSV is useful in treating skin roughness, acne vulgaris (by inhibiting *Propionibacterium acnes*), Herpes simplex virus, psoriasis etc. [2]. Herein, we have selected them to investigate the potential role involved in human keratinocytes’ protection. In addition, we have tried to overcome the limitations of their application due to poor bioavailability and pharmacokinetics by introducing ferrocene into their structure. Ferrocene is an organometallic compound, a ‘sandwich’ complex consisting of an Fe(II) core coordinated to two cyclopentadienyl ligands. The physico-chemical properties of ferrocene (air-, water-, heat-stability, etc.) and its derivatives enable their application in a wide range of fields, including medicinal chemistry [3]. The introduction of a ferrocenyl group into drug or drug-like molecules has a marked effect on molecular properties—modulation of ROS (reactive oxygen species) production, redox activation, and lipophilicity, therefore researchers often explore the enormous potential of this strategy [3]. A novel study by Kim et al. [4] proposed an innovative transdermal drug delivery system using ferrocene-incorporated fibers to enhance the bioavailability and therapeutic efficacy of ascorbyl tetraisopalmitate.

Our previous research points to the advantages of ferrocene derivatives compared to the original polyphenolic substances. The novel ferrocene-containing triacyl derivative of resveratrol (RSV scaffold is retained—esterification of all three hydroxyl groups of RSV with ferrocene introduced into the structure; RF; Table 1) was recently synthesized by our group, and we have determined its beneficial effect on multiple cell viability parameters [5]. In addition to the action of the new resveratrol derivative, object of this study was to examine the effects of a novel ferrocene-containing curcumin analogue (CRC scaffold is modified—one benzylidene group of CRC is replaced by ferrocene; CF; Table 1; the synthesis is presented in our paper as compound 4; [6]) as well as the original compounds—RSV and CRC. So far, research has been directed to antibacterial, antimalarial, antifungal, and anticancer potential of ferrocene-containing compounds [7]. Two properties are essential for their biological activity: (i) the drug delivery design and property of the medicinal cargo, and (ii) the chemical and electrochemical reversibility provided by the ferrocene/ferricenium redox system, serving as an optimal electron reservoir [7]. We hypothesized that ferrocene insertion might enhance properties of RSV and CRC by improving keratinocyte viability and providing protection from radiation- or chemically induced oxidative stress, accompanied by cell death. This study aimed to compare the biological activity of natural and ferrocene-modified polyphenols and to expand knowledge of the application of bioorganometallic compounds in wider areas.

## 2. Materials and Methods

### 2.1. Cell Line and Cell Culture Conditions

The adherent HaCaT cell line was obtained from Cytion, Eppelheim, Germany. The HaCaT cell line was routinely grown in Dulbecco’s Modified Eagle Medium supplemented with 10% (*v*/*v*) fetal bovine serum (atmosphere of 95% air and 5% CO_2_ at 37 °C), obtained from Sigma-Aldrich Co., St. Louis, MO, USA. No antibiotics were used throughout the experiments. Cells were harvested with trypsin/EDTA solution (Sigma-Aldrich Co.) after reaching >70% confluence, diluted to the required density, and seeded in multiwell plates (Nunc, Roskilde, Denmark), depending on the experiments performed.

### 2.2. Cell Viability Determined with MTT and Trypan Blue Methods

The cytotoxicity endpoints were determined by MTT (3-(4,5-dimethyl-2-thiazolyl)-2,5-diphenyl-2H-tetrazolium bromide) assay and Trypan blue method [8]. For the MTT assay, cells were seeded in 96-well transparent flat-bottomed polystyrene plates at an initial concentration of 2.5 × 10^5^ cells mL^−1^ (100 μL/well) in growth medium supplemented with RSV, RF, CRC, and CF at concentrations of 5–100 μM. Concentrations were chosen based on our previously conducted research—reported cytotoxicity thresholds in HaCaT and other cell lines [5,6]. RF and CF were synthesized in the Laboratory for Organic Chemistry at the University of Zagreb Faculty of Food Technology and Biotechnology, Zagreb, Croatia, while RSV and CRC were purchased from Sigma-Aldrich. After 48 h of incubation at 37 °C, cell viability was determined using the MTT assay [8]. The absorbance was measured at 540 nm (Cary Eclipse spectrofluorimeter, Varian, Palo Alto, CA, USA). For the Trypan blue method, the cells were seeded in 6-well plates at an initial concentration of 2 × 10^4^ cells mL^−1^ (4 mL/well). After overnight incubation at 37 °C, the cells were treated with RSV, RF, CRC, and CF at different concentrations (5–100 μM). Cell viability was determined 48 h after treatment using the Trypan blue method [8]. The stock solution of RSV was prepared in EtOH, while CRC and ferrocenyl analogues require DMSO due to higher lipophilicity. All stock solutions were stored at −20 °C until use. To ensure that exposure to the solvents did not affect cell viability, cells in the control samples were exposed to the same concentration of DMSO or EtOH (1% of total medium *v*/*v*) and considered as 100% viable cells. Cell viability is expressed as the percentage of treated cells compared to control cells. For each concentration tested, 3–15 independent biological samples were analyzed.

### 2.3. Intracellular ROS Determination Induced with tert-Butyl Hydroperoxide or UV

Two approaches were used for intracellular ROS induction. The first approach involved pretreatment of HaCaT cells with RSV, RF, CRC, and CF and, after overnight incubation, treatment with 50 μM *t*BHP (*tert*-butyl hydroperoxide). The second approach included pretreatment of the cells with CRC and CF, and after overnight incubation, exposure of the cells to UV (ultraviolet)-C light (15 W; 55 V; 254 nm; PURITEC HNS Linear Lamps, HNS 15 W G13, Osram GmbH, Munich, Germany) for 3 h. In brief, HaCaT cells were seeded at a concentration of 2.5 × 10^5^ cells mL^−1^ in black 96-well plates (100 µL/well) in growth medium and supplemented with RSV, RF, CRC, and CF at concentrations 2.5–50 μM. The DCFDA/H2DCFDA (2′,7′-dichlorofluorescin diacetate/2′,7′-dichlorodihydrofluorescein diacetate) assay was performed according to the manufacturer’s protocol with minor modifications (Cellular ROS Assay Kit Cat. No. ab113851, Abcam, Cambridge, UK). The intracellular ROS level was determined by measuring the fluorescence intensity (λ_ex/em_ = 485/535 nm; Cary Eclipse spectrofluorimeter). Cells treated with the same volume of DMSO or EtOH in which RSV, RF, CRC, and CF were dissolved, and cells treated with 50 µM *t*BHP served as controls. In UV experiments, cells treated with the solvent (DMSO) and exposed to UVR (ultraviolet radiation) were considered as control samples. For each concentration tested, 3–8 independent biological samples were analyzed.

### 2.4. Cytofluorimetric Analysis

#### 2.4.1. Apoptosis Evaluation

The Muse™ Annexin V & Dead Cell Kit (Cat. No. MCH100105, Millipore, Merck Darmstadt, Germany) was used to determine the percentage of HaCaT cells in four different cell populations (live, early apoptotic, late apoptotic, and dead) after treatment of cells with RSV, RF, CRC, and CF (5–100 μM; for 48 h) according to the manufacturer’s protocol. A detailed protocol was elucidated in Murati et al. [9]. The cells were analyzed using the Muse™ Cell Analyzer (Luminex, Austin, TX, USA). For each concentration tested, 3–7 independent biological samples were analyzed.

#### 2.4.2. Autophagy Evaluation

The Muse™ Autophagy LC3-antibody based kit (Cat. No. MCH200109, Millipore) was used to determine the induction of autophagy in HaCaT cells after treatment with RSV, RF, CRC, and CF (5–100 μM) with some minor protocol modifications. The experimental procedure for both analyses was set up as for cell viability determination by the Trypan blue method. Autophagy induction data were read based on the fluorescence intensity of the sample (using Muse™ Cell Analyzer). For each concentration tested, 3–8 independent biological samples were analyzed.

### 2.5. Statistical Analysis

The statistical analysis was performed using JASP software version 0.17.1 (University of Amsterdam, The Netherlands). All data are presented as means ± SEM. Differences between groups were assessed using one-way ANOVA, followed by Tukey’s post hoc test (*p* < 0.05 was considered statistically significant).

## 3. Results

### 3.1. Effect of Resveratrol (RSV), Ferrocene-Containing Triacyl Derivative of Resveratrol (RF), Curcumin (CRC) and Ferrocene-Containing Curcumin Analogue (CF) on HaCaT Cell Proliferation and Viability

**Figure 1:**
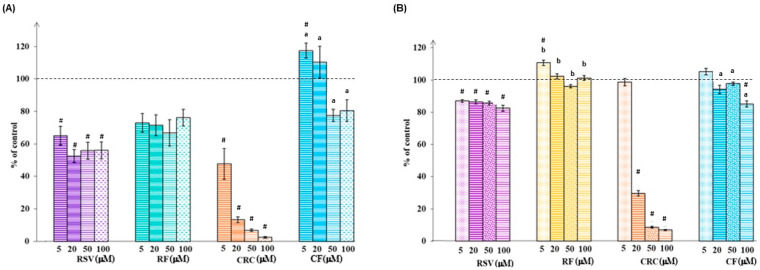
HaCaT cell (human keratinocytes) proliferation after incubation with 5–100 µM resveratrol (RSV), ferrocene-containing triacyl derivative of resveratrol (RF), curcumin (CRC), or ferrocene-containing curcumin analogue (CF) for 48h, determined by Trypan Blue exclusion method (**A**) and MTT method (**B**). Control for RSV—cells treated with ethanol (EtOH); control for RF, CRC, and CF—cells treated with dimethyl sulfoxide (DMSO); viability of cells in control samples is considered as 100%. Data (*n* = 3—15 independent biological samples) are presented as a percentage of the respective control ± SEM. Statistically significant difference (one-way ANOVA, followed by Tukey’s post hoc test; *p* < 0.05) compared to: ^#^ respective control; ^a^ CRC vs. CF; ^b^ RSV vs. RF (comparison of corresponding doses).

RSV and CRC, as well as RF and CF (5–100 µM) were screened for their basic effects on cell proliferation and viability (Figure 1, Table 2). To achieve these endpoints, Trypan Blue and MTT reduction assay were applied. MTT—a highly reproducible and widely used method, measures the dysfunction of the mitochondrial electron transport chain as an indicator of metabolic activity and cell viability, while the TB exclusion test discriminates and measures the number of viable and dead cells based on cell membrane integrity [8]. The summarized results of RSV, RF, CRC, and CF action on the human keratinocyte proliferation and viability after comprehensive in vitro screening are shown in Figure 1A,B.

Both synthetic ferrocene-modified compounds—RF and CF—showed significantly lower antiproliferative activity (Figure 1A and Figure S1—comprehensive statistical analysis, Table 2), and cell survival was markedly pronounced (Figure 1A,B) compared to RSV or CRC-treated cells.

CRC exerted the highest cytotoxicity among tested compounds (IC_50_ = 11.03 µM and 18.91 µM determined by Trypan Blue and MTT assay, respectively, Table 2) cell viability was almost completely impaired in doses ≥ 50 µM (Figure 1A,B), while a statistically significant inhibitory effect (*p* < 0.05) on cell growth was observed even at lower concentrations (≥5 µM) (Figure 1A). RSV exhibited noticeably milder cytotoxicity compared to CRC, which was significant in concentrations ≥ 20 µM (*p* < 0.05), regardless of the method used (Figure 1A and Figure S1).

### 3.2. Effects of Resveratrol (RSV), Curcumin (CRC) or Their Ferrocene Derivatives/Analogues (RF or CF) on ROS Level and Oxidative Stress in HaCaT Cells Induced by tert-Butyl Hydroperoxide (tBHP) or UV Treatment

**Figure 2:**
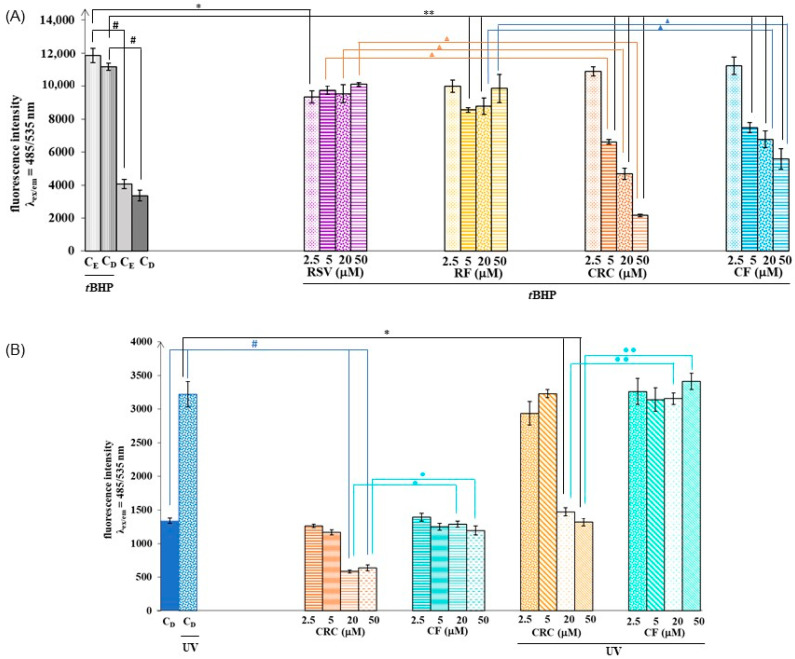
Effect of 2.5–50 µM resveratrol (RSV), ferrocene-containing triacyl derivative of resveratrol (RF), curcumin (CRC), or ferrocene-containing curcumin analogue (CF) on ROS (reactive oxygen species) formation in 50 μM *t*BHP (*tert*-butyl hydroperoxide)-treated (**A**) or UV (ultraviolet)-treated (**B**) HaCaT (human keratinocytes) cells. Control for RSV—cells treated with ethanol (EtOH) (C_E_); control for RF, CRC, and CF—cells treated with dimethyl sulfoxide (DMSO) (C_D_); control for ROS induction—control cells treated with solvent and exposed to ROS inductor (*t*BHP or UV). Data (*n* = 3–8; independent biological samples) are presented as mean ± SEM. Statistically significant difference (one-way ANOVA, followed by Tukey’s post hoc test; *p* < 0.05): (**A**) ^#^ control cells treated with *t*BHP vs. untreated cells (C_E_*/*C_D_); * cells preincubated with RSV and treated with *t*BHP vs. control cells treated with *t*BHP only; ** cells preincubated with RF, CRC or CF and treated with *t*BHP vs. control cells treated with *t*BHP only; ^▲ ^cells preincubated with RSV and treated with *t*BHP vs. cells preincubated with CRC and treated with *t*BHP; ^▲ ^cells preincubated with RF and treated with *t*BHP vs. cells preincubated with CF and treated with *t*BHP. (**B**) ^#^ untreated cells (C_D_) vs. control cells treated with UV (to determine whether UV statistically significant increases ROS) or untreated cells (CD) vs. CRC-treated cells (to determine whether CRC statistically significant influence endogenous ROS formation); * control cells treated with UV vs. cells preincubated with CRC or CF and treated with UV; ^● ^CRC-treated cells vs. CF-treated cells; ^●● ^cells preincubated with CRC and treated with UV vs. cells preincubated with CF and treated with UV.

One of the aims of our study was to determine the effect of the polyphenols RSV and CRC, as well as their ferrocene derivatives RF and CF, on oxidative stress induced by *t*BHP or UV exposure in HaCaT cells. The results of the effect of RSV, RF, CRC, and CF on the ROS level induced by *t*BHP are shown in Figure 2A. All four compounds showed a beneficial effect on the reduction of ROS induced by *t*BHP, with CRC and its synthetic analogue CF having the most pronounced effect. Considering the demonstrated positive results accomplished with CRC and CF, in further experiments, we focused on trials that included only these two compounds. In UV-induced ROS experiments (Figure 2B), CRC had a protective effect on HaCaT cells (in doses ≥ 20 µM), while such an expected outcome was not determined after the application of CF. CRC also significantly reduced endogenous ROS production (in doses ≥ 20 µM, *p* < 0.05) (Figure 2B and Figure S2—comprehensive statistical analysis).

It is important to note that CRC also exhibited a significant antiproliferative effect and that the viability of HaCaT cells was extremely low at high doses of CRC (Figure 1A,B), which could be the reason for the detection of a low fluorescence signal of ROS (therefore, caution is needed when interpreting these results). In contrast to CRC, the ferrocene analogue CF did not reduce cell viability (Figure 1A,B), and the lowering of the ROS level induced by *t*BHP (Figure 2A) achieved by its action can be considered accurate and protective.

### 3.3. The Involvement of Natural Polyphenols (RSV or CRC) or Their Ferrocene Derivatives/Analogues (RF or CF) in Molecular Mechanisms Inducing Cell Death in HaCaT Cell Culture

#### 3.3.1. Apoptosis

**Figure 3:**
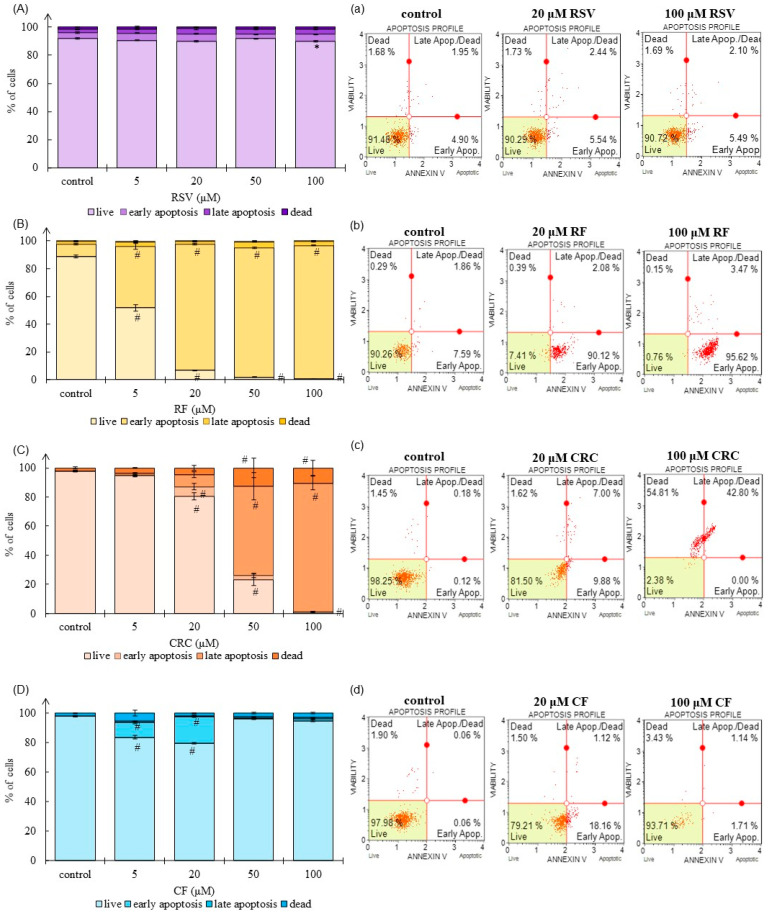
Apoptosis/necrosis cell death evaluation in HaCaT cells (human keratinocytes) after exposure to 5–100 μM resveratrol (RSV; (**A**,**a**)), ferrocene-containing triacyl derivative of resveratrol (RF; (**B**,**b**)), curcumin (CRC; (**C**,**c**)) or ferrocene-containing curcumin analogue (CF; (**D**,**d**)) for 48 h by Muse™ Cell Analyzer. Control for RSV—cells treated with ethanol (EtOH); control for RF, CRC, and CF—cells treated with dimethyl sulfoxide (DMSO). Data (*n* = 3–7; independent biological samples) are presented as a percentage of live, early, and late apoptotic, and dead cells in the population; mean ± SEM. Statistically significant difference (one-way ANOVA, followed by Tukey’s post hoc test; *p* < 0.05; all cell populations—live, early apoptotic, late apoptotic, dead cells, were compared to respective cell populations in control): (**A**) * control (EtOH) vs. 5–100 μM RSV; (**B**) # control (DMSO) vs. 5–100 μM RF; (**C**) # control (DMSO) vs. 5–100 μM CRC; (**D**) # control (DMSO) vs. 5–100 μM CF.

Flow cytometry analyses were performed to examine levels of apoptosis/necrosis in human keratinocytes after treatment with CRC, RSV, or their ferrocene derivatives/analogues (RF or CF). The induction of apoptosis was evidenced by phosphatidylserine externalization using a well-known marker, annexin V. Among the tested compounds, CRC induced cell death to the greatest extent, and at the highest concentration, viability was only 1.08%, while the proportion of cells in late apoptosis was 88.34% and necrosis 10.52% (Figure 3C,c). CRC induced a dose-dependent shift toward late apoptosis. RSV was less cytotoxic compared to CRC and did not significantly affect cell viability, except at the 100 µM dose, where cell survival (proportion of viable cells in the culture) was significantly lower (*p* < 0.05; Figure 3A,a, Supplementary Figure S3). Early apoptosis was stimulated by the ferrocene derivative RF, and the majority of cells remained in this fraction after treatment with RF doses ≥ 5 µM (Figure 3B,b). CF induced cell death significantly less compared to all compounds tested (Figure 3D,d), and these results were consistent with those obtained in basal cytotoxicity assays (Figure 1A,B).

#### 3.3.2. Autophagy

**Figure 4:**
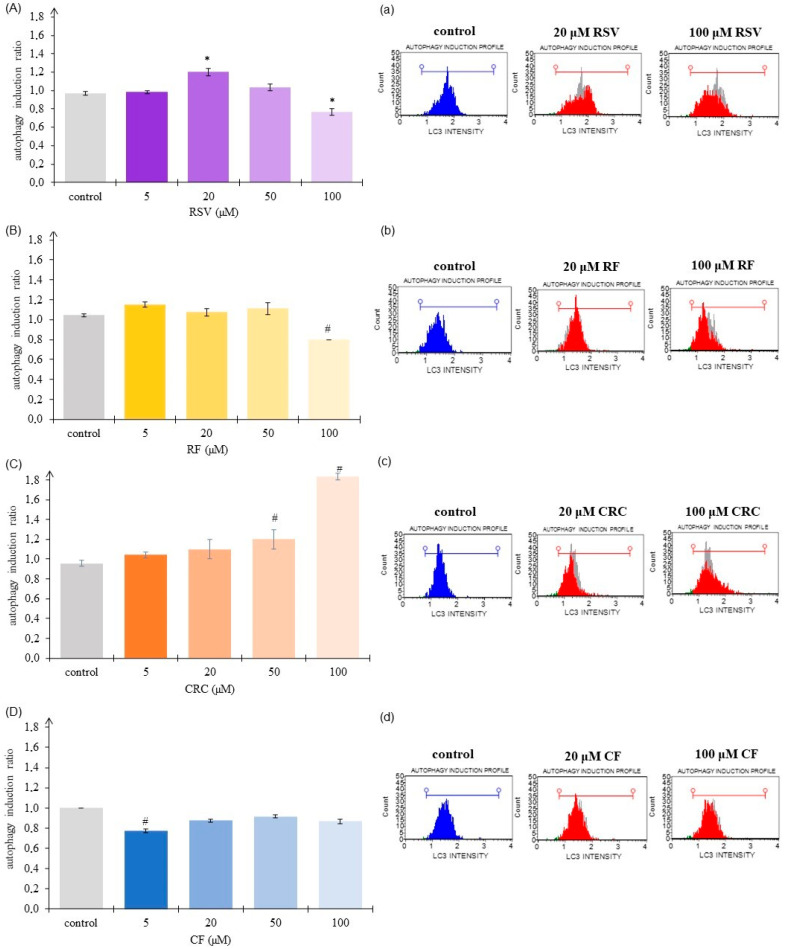
Autophagy cell death evaluation in HaCaT cells (human keratinocytes) after exposure to 5–100 μM resveratrol (RSV; (**A**,**a**)), ferrocene-containing triacyl derivative of resveratrol (RF; (**B**,**b**)), curcumin (CRC; (**C**,**c**)) or ferrocene-containing curcumin analogue (CF; (**D**,**d**)) for 48 h by Muse™ Cell Analyzer. Control for RSV—cells treated with ethanol (EtOH); control for RF, CRC, and CF—cells treated with dimethyl sulfoxide (DMSO). Data (*n* = 3–8; independent biological samples) are presented as autophagy induction ratio (test sample fluorescence relative to control); mean ± SEM. Statistically significant difference (one-way ANOVA, followed by Tukey’s post hoc test; *p* < 0.05): (**A**) * control (EtOH) vs. 5–100 μM RSV; (**B**) # control (DMSO) vs. 5–100 μM RF; (**C**) # control (DMSO) vs. 5–100 μM CRC; (**D**) # control (DMSO) vs. 5–100 μM CF.

Quantification of LC3-positive (fluorescent-marked) structures in the cells was the endpoint in the determination of autophagy by flow cytometry after treatment of HaCaT cell culture with CRC, RSV, RF, or CF. As LC3-II is specifically associated with autophagosomes and autolysosomes (in the absence of conditions stimulating LC3-associated phagocytosis), quantification of LC3-positive puncta is considered to be a reliable method for assessing autophagic flux [10]. Our research showed that the polyphenols CRC and RSV induce autophagy to a greater extent than their ferrocene-modified derivatives (RF and CF). Comparing the results for all tested compounds (Figure 4, Supplementary Figure S4), it is noticeable that the highest autophagy induction ratio was determined for CRC at the highest dose and that the shift toward autophagy was clearly dose-dependent (Figure 4C,c).

Conversely, after the introduction of ferrocene into the structure of CRC, the newly synthesized compound CF even reduced autophagic degradation activity (Figure 4D,d). Significantly (*p* < 0.05) lower autophagy-related LC3 protein was also quantified at the highest dose of RF (Figure 4B,b).

## 4. Discussion

Polyphenols as functional ingredients have attracted substantial attention from the pharmaceutical and cosmetic industries in recent years. Scientific research related to CRC and RSV has increased considerably in the last decades, with 43.536 records for the term (Field—topic) “curcumin” in the Web of Science Core Collection database and 33.386 records for “resveratrol” (on 12 November 2025). RSV (Table 1) is a stilbene phytoalexin synthesized by a variety of plants (e.g., *Vitis vinifera*—grapes, *Arachis hypogaea*—peanut, *Pistacia vera* L.—pistachio, *Theobroma cacao* L.—cocoa [5]) in response to fungi infections or UVR, while CRC (diferuloylmethane; Table 1) is an active ingredient of the perennial herb *Curcuma longa*, also known as turmeric. Both polyphenols have been described as anti-inflammatory, antioxidant, anti-aging, tissue-repairing, and chemopreventive compounds; however, the mode of action for them, in many aspects, is still unclear [11,12]. In addition, numerous clinical trials investigating CRC and RSV (listed on ClinicalTrials.gov [13] on 12 November 2025) are ongoing or completed—220 for RSV and 371 for CRC. The majority of human clinical trials have described various aspects of CRC and RSV pharmacodynamics and pharmacokinetics parameters, reaching a consensus that they are generally well tolerated, but have poor bioavailability [14,15]. In order to overcome these obstacles, numerous approaches have been proposed, such as different encapsulation techniques, the use of prodrugs, and various structural modifications of polyphenols. Recently, our group has demonstrated a beneficial effect of the novel resveratrol derivative—RF (Table 1) on multiple cellular endpoints in ovarian cell culture, as well as the protective effect of RSV against ROS-induced cytotoxicity associated with age-related and chronic diseases (including Alzheimer’s disease and dementia, cancer, diabetes mellitus, and cardiopulmonary disease) [5]. Following these positive results obtained, in this study, we examined the biological activity of ferrocene-modified polyphenols (RF and CF; Table 1) in human skin cells. HaCaT cell line (human keratinocytes) has acceptable predictive value for the safety evaluation of compounds after skin exposure [16]. Human primary keratinocytes isolated from the skin of healthy donors represent the most suitable model for physiological and biochemical studies of the skin. However, this in vitro model is limited due to their relatively short lifespan, difficulties in culturing, and genome editing. In contrast, immortalized human keratinocyte cell lines, as HaCaT, are easy to culture and manipulate genetically, thus providing stable and reproducible conditions for studying the intracellular signaling pathways in skin biology [17].

Several endpoints in HaCaT cells, commonly used to explore skin health, epidermal homeostasis, and associated pathologies, were assessed using basal cytotoxicity experiments in the first phase of our research. For systematic analysis of cell proliferation and viability, two validated bioassays were chosen: the TB exclusion method (Figure 1A) and the MTT reduction assay (Figure 1B). Statistically significant (*p* < 0.05) cytotoxicity was confirmed for both polyphenols (RSV in doses ≥ 5 µM and CRC in doses ≥ 20 µM) by both methods used, with CRC showing more pronounced effects in cell viability reduction. After the structural modifications of RSV and CRC with ferrocene, the novel synthetic compounds RF and CF no longer had such a marked antiproliferative effect (Figure 1, Table 2). At certain doses, CF improved cell viability, and cell proliferation reached and even exceeded the values obtained in the control (untreated) sample (Figure 1A,B). In CF (Table 1), one benzylidene group of CRC is replaced by ferrocene, and the β-diketone moiety is preserved, which resulted in an altered structure that proved to be less cytotoxic in human skin cell culture. Most of the literature data focuses on the antitumor effect of CRC analogues, based on the inhibition of cancer cell growth, anti-angiogenesis, and stimulation of cell death, mainly through apoptosis or autophagy [7,18,19]. Unlike recent scientific research, the aim of this study was the selection of those compounds that have a beneficial effect on cellular health and viability, while retaining antioxidant activity, which is particularly important in the case of skin tissue that is constantly exposed to free radicals’ damage and oxidative stress.

Extrinsic skin damage develops due to multiple factors, including ionizing radiation, severe physical and psychological stress, alcohol intake, poor nutrition or overeating, environmental contaminants, and exposure to UVR. It is estimated that among all these factors, UVR contributes up to 80%. The primary mechanism by which UVR triggers molecular responses in human skin is via photochemical generation of ROS, particularly the formation of superoxide anion (O_2_^•−^), hydrogen peroxide (H_2_O_2_), hydroxyl radical (^•^OH), and singlet oxygen (^1^O_2_) [20].

In the present study, it was evidenced that pretreatment of human keratinocytes with the RSV, CRC, RF, or CF protects cells from *t*BHP- or UV-induced injury and oxidative damage (Figure 2A,B). All four compounds showed a beneficial effect on the reduction of ROS induced by *t*BHP (Figure 2A), but among them, CRC and its synthetic analogue CF can be singled out for their effectiveness. At doses ≥ 5 µM, both CRC and CF showed statistically significant activity (*p <* 0.05) related to the lowering of ROS levels. Literature data regarding CRC and RSV support the results of this research and are extensive in claiming the antioxidant effects of these polyphenols [21]. It should also be emphasized that the synthetic derivatives RF and CF significantly reduced the ROS induced by *t*BHP (Figure 2A), while they did not negatively affect cell viability and proliferation (Figure 1A,B), which marks them as potentially protective substances against oxidative stress. In UV experiments (Figure 2B), we further focused on trials that included CRC and CF, considering promising results obtained in the research with *t*BHP-stimulated ROS production (Figure 2A). Here, the efficacy of CRC was clearly observed (although the pronounced antiproliferative effect of CRC should be taken into account when interpreting the results; Figure 1A,B), while at the same dose range, CF did not exhibit significant antioxidant activity (Figure 2B). The effectiveness of the tested compounds (RSV, CRC, RF, or CF) was shown to be greater when oxidative stress resulted from exposure to *t*BHP, and the results show the potential of ferrocene-modified polyphenols in mitigating oxidative damage in human keratinocytes. Other scientific studies have also reported on the antioxidant capacities of ferrocenyl-substituted CRC derivatives. Li and Liu [22] pointed out in their research the abilities of FCU (1,7-bis(p-hydroxy-m-methoxyphenyl)-4-ferrocenylidene-hepta-1,6-diene-3,5-dione), FFT (1-(p-hydroxy-m-methoxyphenyl)-3-hydroxy-7-ferrocenyl-hepta-1,4,6-trien-5-one), and FDZ (1-(p-hydroxy-m-methoxyphenyl)-5-ferrocenyl-penta-1,4-dien-3-one) to inhibit AAPH (2,2′-azobis (2-amidinopropane) hydrochloride)-induced oxidation of DNA in higher extent than CRC, feruloylacetone, and dehydrozingerone. FCU, FFT, and FDZ also exhibited protective effects on Cu^2+^/GSH-induced oxidation of DNA. Thus, authors concluded that the introduction of the ferrocenyl group into CRC enhances the antioxidant abilities of FCU, FFT, and FDZ in certain cases [22].

Potential applications of ferrocene as a structural feature in antioxidants were summarized in the review article by Liu (2011) [23]. The large conjugated ferrocenyl derivatives were found to be beneficial for the single electron to dispense among the entire molecule while forming radicals, and to increase the antioxidant capacity of the whole molecule. Taking into consideration the importance of antioxidative compounds to maintain health and to cure ROS-induced diseases (in the pathology of cancer, cardiovascular diseases, diabetes, neurological disorders—Alzheimer’s and Parkinson’s disease, Down syndrome, psychiatric diseases—depression, schizophrenia, bipolar disorder, renal disease, lung diseases—chronic pulmonary obstruction, lung cancer, and aging), designing antioxidants with novel structures is an attractive research field in organic and medicinal chemistry [23,24].

To clarify the cell death mechanism mediated by RSV, CRC, RF, or CF, flow cytometric analysis was performed (Figure 3 and Figure 4). Among all the tested compounds, CRC induced apoptosis to the greatest extent, and at the highest CRC concentration (100 µM), the proportion of cells in early and late apoptosis was 88.40% (Figure 3C,c). Proapoptotic and antiproliferative effects of CRC are well documented [25,26] and the possibilities of using CRC in the treatment of chronic skin diseases characterized by keratinocyte hyperproliferation and abnormal differentiation (as psoriasis) are examined [27]. RSV was less cytotoxic, and the level of cell death through necrotic or apoptotic pathways did not exceed 11% even at 100 µM RSV (Figure 3A,a). In our previous study [28], we also showed that RSV reduced apoptotic death of *ortho*-PCB-treated cells. After ferrocene introduction into the structure of RSV and CRC, novel compounds showed altered biological activity (Figure 3B,b,D,d). Early apoptosis was stimulated by the ferrocene derivative RF, while treatment with CF had almost no effect on cell viability, and these results were consistent with those obtained by applying basal cytotoxicity assays (Figure 1A,B).

Autophagy was another type of cell death that we investigated after exposure of the cells to the tested compounds. RSV had a dual effect on the induction of autophagy (Figure 4A,a), stimulating it at certain doses (20 µM; *p* < 0.05) and reducing it at others (100 µM; *p* < 0.05). The most prominent autophagy induction ratio was determined for CRC in a concentration of 100 µM (Figure 4C,c), and the autophagic flux after treatment of cells with CRC, was clearly dosage-dependent. The order of the autophagy induction ratio (from highest to lowest) at 100 µM concentration was: CRC > CF > RF ≈ RSV. Molecular mechanisms of CRC-induced autophagy are diverse and mostly studied in cancerous cell cultures. Specific signaling pathways are activated in different tumor cells, namely the knockdown of the PI3K/Akt/mTOR (phosphoinositide 3-kinase–AKT-mammalian target of rapamycin) signaling pathway, induction of adenosine monophosphate (AMP)-activated protein kinase (AMPK) pathway and ERK1/2 (extracellular signal-regulated kinases 1 and 2) pathway, formation of ROS, as well as development of endoplasmic reticulum (ER) stress [29]. Autophagy is a lysosomal degradation pathway for cellular constituents and organelles, an essential process required for cellular homeostasis. However, autophagy can also lead to a non-apoptotic form of programmed cell death called autophagy-associated cell death. The potential of CRC or RSV to initiate autophagic processes should therefore be considered from different aspects, and their use as therapeutics should be adjusted depending on the targeted and desired effects linked to specific diseases. RSV derivative—RF at the lowest administered dose (5 µM) slightly induced autophagy, while treatment with CF even led to a decrease in the autophagy induction ratio in HaCaT cells (Figure 4B,D).

This study determined the effects of natural and ferrocene-modified nutraceuticals at the cellular level using the HaCaT model. In addition to keratinocytes, research related to skin protection should be carried out on other skin cell cultures, such as those derived from melanocytes, sweat glands, and microvascular endothelial cells, etc. Other than these toxicodynamic parameters, the toxicokinetics of the investigated compounds should also be assessed. It would be particularly important to determine their bioavailability and the actual amount of compound that penetrates into the cells. Appropriate in vivo models would provide insight into overall ADMET. This research is a good starting point for the development of new potential ferrocene-modified nutraceuticals.

## 5. Conclusions

Ferrocene-modified novel compounds RF and CF affected cell viability to a lesser extent compared to RSV and CRC. Altered biological activity was also observed for apoptosis and autophagy induction, with a lower ratio of cell death related to RF and CF. Oxidative stress is reduced by the use of nutraceuticals (RSV and CRC). Ferrocene-modified polyphenols also effectively reduced chemically induced ROS, while CRC was the most prominent after UV treatment of keratinocytes. By retaining antioxidant activity, RF and CF can potentially protect skin cells from degenerative processes, aging, ROS-induced skin cellular senescence, inflammation, and cancer. It is important to note that studies related to ferrocene-modified polyphenols should be conducted using in vivo models and more comprehensively with deeper analysis of intracellular events, resulting in possible clinical trials. Therefore, novel research in the field of organometallic chemistry/ferrocene-modified polyphenols is advisable, not only in the development of anticancer agents, but also in the selection of compounds that may have a beneficial and protective effect on cell viability and health, reducing the possibility of developing chronic degenerative diseases.

## Figures and Tables

**Table 1:** Polyphenols and their ferrocene analogues used in research.

Chemical Structure	Compound, Abbreviation	Name, CAS RN	Molecular Formula	Molecular Weight (g mol^−1^)
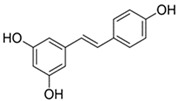	resveratrol, RSV	3,4′,5-trihydroxy-*trans*-stilbene CAS RN 501-36-0	C_14_H_12_O_3_	228.25
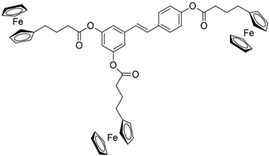	ferrocene-containing triacyl derivative of resveratrol, RF	*trans*-3,5,4′-tri(4-ferrocenylbutanoyloxy)-stilbene	C_56_H_54_Fe_3_O_6_	990.58
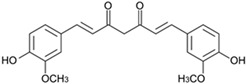	curcumin, CRC	(1*E*,6*E*)-1,7-bis(4-hydroxy-3-methoxyphenyl)-1,6-heptadiene-3,5-dione CAS RN 458-37-7	C_21_H_20_O_6_	368.39
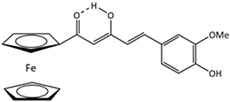	ferrocene-containing curcumin analogue, CF	(*E*)-5-(4-hydroxy-3-methoxyphenyl)-1-ferrocenylpent-4-ene-1,3-dione	C_22_H_20_FeO_4_	404.24

**Table 2:** IC values (µM) for HaCaT cells (human keratinocytes) following exposure to resveratrol (RSV), ferrocene-containing triacyl derivative of resveratrol (RF), curcumin (CRC), or ferrocene-containing curcumin analogue (CF) for 48 h, based on dose–response curves as derived from Trypan blue exclusion method and MTT assay.

IC Values (µM)	Trypan Blue Method				MTT Method			
	RSV	RF	CRC	CF	RSV	RF	CRC	CF
**IC_20_**	3.22	10.78	n.d.	75.40	n.d.	n.d.	7.28	120.58 *
**IC_50_**	40.34	n.d.	11.03	n.d.	n.d.	n.d.	18.91	208.04 *
**IC_80_**	n.d.	n.d.	26.44	n.d.	n.d.	n.d.	34.04	272.61 *

n.d. (not determined)—values cannot be calculated from the corresponding equations of the interpolated curves. * values are outside the range of the tested doses (>100 µM).

## Data Availability

The original contributions presented in this study are included in the article/Supplementary Material. Further inquiries can be directed to the corresponding author.

## Supplementary Materials

The following supporting information can be downloaded at: https://www.mdpi.com/article/10.3390/pharmaceutics17121511/s1, Figure S1: HaCaT cell (human keratinocytes) proliferation after incubation with 5–100 µM resveratrol (RSV), ferrocene-containing triacyl derivative of resveratrol (RF), curcumin (CRC), or ferrocene-containing curcumin analogue (CF) for 48h, determined by Trypan Blue exclusion method (A) and MTT method (B). Control for RSV—cells treated with ethanol (EtOH); control for RF, CRC, and CF—cells treated with dimethyl sulfoxide (DMSO). Data (n = 3–15; independent biological samples) are presented as a percentage of the respective control ± SEM. Statistically significant difference (one-way ANOVA, followed by Tukey’s post hoc test; *p* < 0.05) compared to: ^*^control (EtOH), ^#^control (DMSO), ^a^RSV5 vs. RF5, CRC5, CF5; ^b^RSV20 vs. RF20, CRC20, CF20; ^c^RSV50 vs. RF50, CRC50, CF50; ^d^RSV100 vs. RF100, CRC100, CF100; ^e^RF5 vs. CRC5, CF5; ^f^RF20 vs. CRC20, CF20; ^g^RF50 vs. CRC50, CF50; ^h^RF100 vs. CRC100, CF100; ^I^CRC5 vs. CF5; ^j^CRC20 vs. CF20; ^k^CRC50 vs. CF50; ^l^CRC100 vs. CF100.; Figure S2: Effect of 2.5–50 µM resveratrol (RSV), ferrocene-containing triacyl derivative of resveratrol (RF), curcumin (CRC), or ferrocene-containing curcumin analogue (CF) on ROS (reactive oxygen species) formation in 50 μM *t*BHP (tert-butyl hydroperoxide)-treated (A) or UV (ultraviolet)-treated (B) HaCaT (human keratinocytes) cells. Control for RSV—cells treated with ethanol (EtOH) (CE); control for RF, CRC, and CF—cells treated with dimethyl sulfoxide (DMSO) (CD). Data (n = 3–8; independent biological samples) are presented as mean ± SEM. Statistically significant difference (one-way ANOVA, followed by Tukey’s post hoc test; *p* < 0.05): (A) ^*^CE vs. *t*BHP + CE; ^#^CD vs. CD + *t*BHP; *t*BHP compared samples: ^&^CE vs. RSV2.5–50; ^$^CD vs. RF2.5–50, CRC2.5–50, CF2.5–50; ^b^RSV5 vs. RF5, CRC5, CF5; ^c^RSV20 vs. RF20, CRC20, CF20; ^d^RSV50 vs. RF50, CRC50, CF50; ^g^RF20 vs. CRC20, CF20; ^h^RF50 vs. CRC50, CF50; ^k^CRC20 vs. CF20; ^l^CRC50 vs. CF50. not significant: RSV2.5 vs. RF2.5, CRC2.5, CF2.5; RF2.5 vs. CRC2.5, CF2.5; RF5 vs. CRC5, CF5; CRC2.5 vs. CF2.5; CRC5 vs. CF5. (B) ^#^CD vs. UV + CD, CRC2.5–50, CF2.5–50; ^*^UV + CD vs. UV + CRC2.5–50, UV + CF2.5–50; ^a^CRC2.5 vs. CF2.5, UV + CRC2.5, UV + CF2.5; ^b^CRC5 vs. CF5, UV + CRC5, UV + CF5; ^c^CRC20 vs. CF20, UV + CRC20, UV + CF20; ^d^CRC50 vs. CF50, UV + CRC50, UV + CF50; ^e^CF2.5 vs. UV + CRC2.5, UV + CF2.5; ^f^CF5 vs. UV + CRC5, UV + CF5; ^g^CF20 vs. UV + CRC20, UV + CF20; ^h^CF50 vs. UV + CRC50, UV + CF50; ^k^UV + CRC20 vs. UV + CF20; ^l^UV + CRC50 vs. UV + CF50. not significant: UV + CRC2.5 vs. UV + CF2.5; UV + CRC5 vs. UV + CF5; Figure S3: Representative dot plots obtained by cytofluorimetric analysis of apoptosis/necrosis in HaCaT cell culture after exposure to 5–100 μM resveratrol (RSV; A), ferrocene-containing triacyl derivative of resveratrol (RF; B), curcumin (CRC; C) or ferrocene-containing curcumin analogue (CF; D) for 48 h (Muse™ Cell Analyzer). Control for RSV—cells treated with EtOH; control for RF, CRC, and CF—cells treated with DMSO. Lower left quadrant—live cells (7-AAD (-), annexin V (-)), lower right quadrant—early apoptotic cells (7-AAD (-), annexin V (+)), upper right quadrant—late apoptotic/dead cells (7-AAD (+), annexin V (+)), and upper left quadrant—dead cells (7-AAD (+) / annexin V(-)).; Figure S4: Representative histograms obtained by cytofluorimetric analysis of autophagy in HaCaT cell culture after exposure to 5–100 μM resveratrol (RSV; A), ferrocene-containing triacyl derivative of resveratrol (RF; B), curcumin (CRC; C), or ferrocene-containing curcumin analogue (CF; D) for 48 h (Muse™ Cell Analyzer). Control for RSV—cells treated with EtOH; control for RF, CRC, and CF—cells treated with DMSO. Autophagy induction ratio (test sample fluorescence, red histogram, vs. control sample fluorescence, gray histogram) is presented.

## Abbreviations

The following abbreviations are used in this manuscript:

AAPH	2,2′-azobis (2-amidinopropane) hydrochloride
AMP	adenosine monophosphate
AMPK	adenosine monophosphate-activated protein kinase
CF	ferrocene-containing curcumin analogue
CRC	curcumin
DCFDA/H2DCFDA	2′,7′-dichlorofluorescin diacetate/2′,7′-dichlorodihydrofluorescein diacetate
DMSO	dimethyl sulfoxide
ER	endoplasmic reticulum
ERK1/2	extracellular signal-regulated kinases 1 and 2
EtOH	ethanol
FCU	1,7-bis(*p*-hydroxy-*m*-methoxyphenyl)-4-ferrocenylidene-hepta-1,6-diene-3,5-dione
FFT	1-(*p*-hydroxy-*m*-methoxyphenyl)-3-hydroxy-7-ferrocenyl-hepta-1,4,6-trien-5-one
FDZ	1-(*p*-hydroxy-*m*-methoxyphenyl)-5-ferrocenyl-penta-1,4-dien-3-one
MTT	3-(4,5-dimethyl-2-thiazolyl)-2,5-diphenyl-2H-tetrazolium bromide
PCB	polychlorinated biphenyls
PI3K/Akt/mTOR	phosphoinositide 3-kinase–AKT-mammalian target of rapamycin
RF	ferrocene-containing triacyl derivative of resveratrol
ROS	reactive oxygen species
RSV	resveratrol
*t*BHP	*tert*-butyl hydroperoxide
UV	ultraviolet
UVR	ultraviolet radiation
