# Supplementary material for: Curcumin and Resveratrol vs. Ferrocene-Modified Polyphenols: Role in Enhancing Protective Properties in Human Keratinocytes
Source: Pharmaceutics. 2025 Nov 22;17(12):1511. doi: 10.3390/pharmaceutics17121511 (PMC12736313; doi:10.3390/pharmaceutics17121511)
Supplement: Supplementary file 1 [file pharmaceutics-17-01511-s001.zip › pharmaceutics-3961054-supplementary.pdf]

pharmaceutics

MDPI

Article

Supplementary material

Curcumin and Resveratrol vs. Ferrocene-Modified Polyphenols: Role in Enhancing Protective Properties in Human Keratinocytes

Marina Miletić [1], Veronika Kovač [2], Lidija Barišić [2], Alen Supičić [1], Bruno Doskočil [1,3], Irena Landeka Jurčević [4], Jelka Pleadin [5], Branimir Šimić [1], Ivana Kmetič [1,*] and Teuta Murati [1]

1 Laboratory for Toxicology, University of Zagreb Faculty of Food Technology and Biotechnology, Pierotti St. 6, 10000 Zagreb, Croatia; mmiletic@pbf.hr (M.M.); alen.supicic@gmail.com (A.S.); bruno.doskocil@pliva.com (B.D.); branimir.simic2@gmail.com (B.Š.); tmurati@pbf.hr (T.M.)
2 Laboratory for Organic Chemistry, University of Zagreb Faculty of Food Technology and Biotechnology, Pierotti St. 6, 10000 Zagreb, Croatia; vkovac@pbf.hr (V.K.); lidija.barisic@pbf.unizg.hr (L.B.)
3 PLIVA Hrvatska d.o.o., Prilaz Baruna Filipovića 25, 10000 Zagreb, Croatia
4 Laboratory for Food Chemistry and Biochemistry, University of Zagreb Faculty of Food Technology and Biotechnology, Pierotti St. 6, 10000 Zagreb, Croatia; ilandeka@pbf.hr
5 Croatian Veterinary Institute, Savska St. 143, 10000 Zagreb, Croatia; pleadin@veinst.hr
* Correspondence: ikmetic@pbf.hr; Tel.: +385-1-4605097

Content:

Figure S1: HaCaT cell (human keratinocytes) proliferation after incubation with 5–100 μM resveratrol (RSV), ferrocene-containing triacyl derivative of resveratrol (RF), curcumin (CRC), or ferrocene-containing curcumin analogue (CF) for 48h, determined by Trypan Blue exclusion method (A) and MTT method (B). Control for RSV - cells treated with ethanol (EtOH); control for RF, CRC, and CF - cells treated with dimethyl sulfoxide (DMSO). Data (n=3 - 15; independent biological samples) are presented as a percentage of the respective control ± SEM. Statistically significant difference (one-way ANOVA, followed by Tukey's *post hoc* test; $p<0.05$) compared to: [*]control (EtOH), [#]control (DMSO), [a]RSV5 *vs.* RF5, CRC5, CF5; [b]RSV20 *vs.* RF20, CRC20, CF20; [c]RSV50 *vs.* RF50, CRC50, CF50; [d]RSV100 *vs.* RF100, CRC100, CF100; [e]RF5 *vs.* CRC5, CF5; [f]RF20 *vs.* CRC20, CF20; [g]RF50 *vs.* CRC50, CF50; [h]RF100 *vs.* CRC100, CF100; [i]CRC5 *vs.* CF5; [j]CRC20 *vs.* CF20; [k]CRC50 *vs.* CF50; [l]CRC100 *vs.* CF100.

Figure S2: Effect of 2.5–50 μM resveratrol (RSV), ferrocene-containing triacyl derivative of resveratrol (RF), curcumin (CRC), or ferrocene-containing curcumin analogue (CF) on ROS (reactive oxygen species) formation in 50 μM *t*BHP (tert-butyl hydroperoxide)-treated (A) or UV (ultraviolet)-treated (B) HaCaT (human keratinocytes) cells. Control for RSV - cells treated with ethanol (EtOH) (CE); control for RF, CRC, and CF - cells treated with dimethyl sulfoxide (DMSO) (CD). Data (n=3 – 8; independent biological samples) are presented as mean ± SEM. Statistically significant difference (one-way ANOVA, followed by Tukey's *post hoc* test; $p<0.05$): (A) [*]CE *vs.* *t*BHP + CE; [#]CD *vs.* CD + *t*BHP; *t*BHP compared samples: [&]CE *vs.* RSV2.5-50; [$]CD *vs.* RF2.5-50, CRC2.5-50, CF2.5-50; [b]RSV5 *vs.* RF5, CRC5, CF5; [c]RSV20 *vs.* RF20, CRC20, CF20; [d]RSV50 *vs.* RF50, CRC50, CF50; [g]RF20 *vs.* CRC20, CF20; [h]RF50 *vs.* CRC50, CF50; [k]CRC20 *vs.* CF20; [l]CRC50 *vs.* CF50. not significant: RSV2.5 *vs.* RF2.5, CRC2.5, CF2.5; RF2.5 *vs.* CRC2.5, CF2.5; RF5 *vs.* CRC5, CF5; CRC2.5 *vs.* CF2.5; CRC5 *vs.* CF5. (B) [#]CD *vs.* UV + CD, CRC2.5-50, CF2.5-50; [*]UV + CD *vs.* UV + CRC2.5-50, UV + CF2.5-50; [a]CRC2.5 *vs.* CF2.5, UV + CRC2.5, UV + CF2.5; [b]CRC5 *vs.* CF5, UV + CRC5, UV + CF5; [c]CRC20 *vs.* CF20, UV + CRC20, UV + CF20; [d]CRC50 *vs.* CF50, UV + CRC50, UV + CF50; [e]CF2.5 *vs.* UV + CRC2.5, UV + CF2.5; [f]CF5 *vs.* UV + CRC5, UV + CF5; [g]CF20 *vs.* UV + CRC20, UV + CF20; [h]CF50 *vs.* UV + CRC50, UV + CF50; [k]UV + CRC20 *vs.* UV + CF20; [l]UV + CRC50 *vs.* UV + CF50. not significant: UV + CRC2.5 *vs.* UV + CF2.5; UV + CRC5 *vs.* UV + CF5.

Figure S3: Representative dot plots obtained by cytofluorimetric analysis of apoptosis/necrosis in HaCaT cell culture after exposure to 5–100 μM resveratrol (RSV; A), ferrocene-containing triacyl derivative of resveratrol (RF; B), curcumin (CRC; C) or ferrocene-containing curcumin analogue (CF; D) for 48 h (Muse™ Cell Analyzer). Control for RSV - cells treated with EtOH; control for RF, CRC and CF - cells treated with DMSO. Lower left quadrant – live cells (7-AAD (-), annexin V (-)), lower right quadrant – early apoptotic cells (7-AAD (-), annexin V (+)), upper right quadrant – late apoptotic/dead cells (7-AAD (+), annexin V (+)), and upper left quadrant – dead cells (7-AAD (+) / annexin V(-)).

Figure S4: Representative histograms obtained by cytofluorimetric analysis of autophagy in HaCaT cell culture after exposure to 5–100 μM resveratrol (RSV; A), ferrocene-containing triacyl derivative of resveratrol (RF; B), curcumin (CRC; C) or ferrocene-containing curcumin analogue (CF; D) for 48 h (Muse™ Cell Analyzer). Control for RSV - cells treated with EtOH; control for RF, CRC and CF - cells treated with DMSO. Autophagy induction ratio (test sample fluorescence, red histogram, *vs.* control sample fluorescence, gray histogram) is presented.

 https://doi.org/10.3390/pharmaceutics17121511

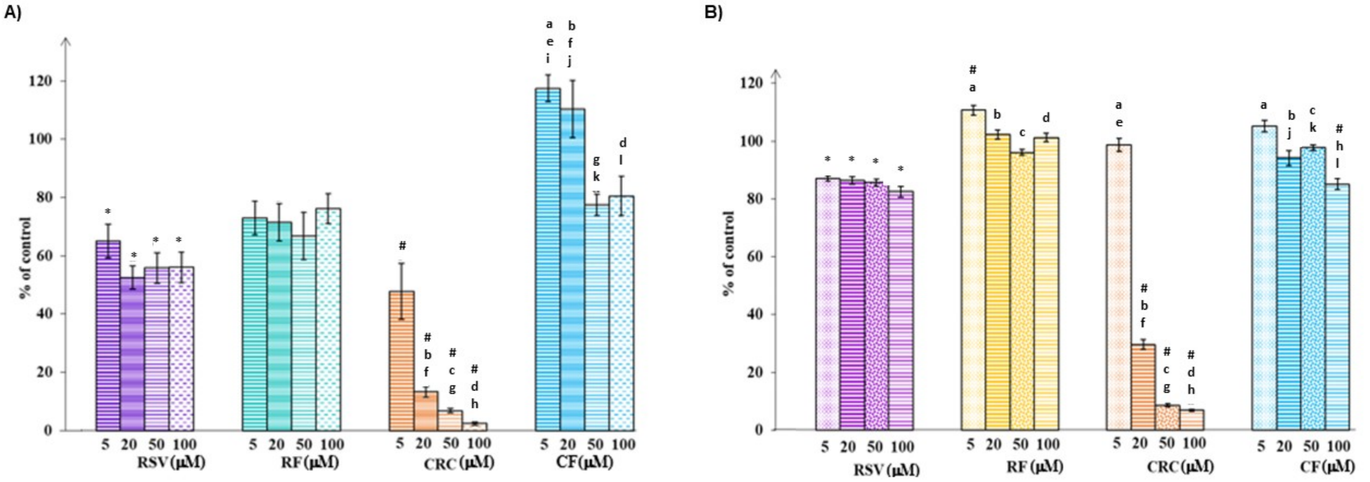

Figure S1

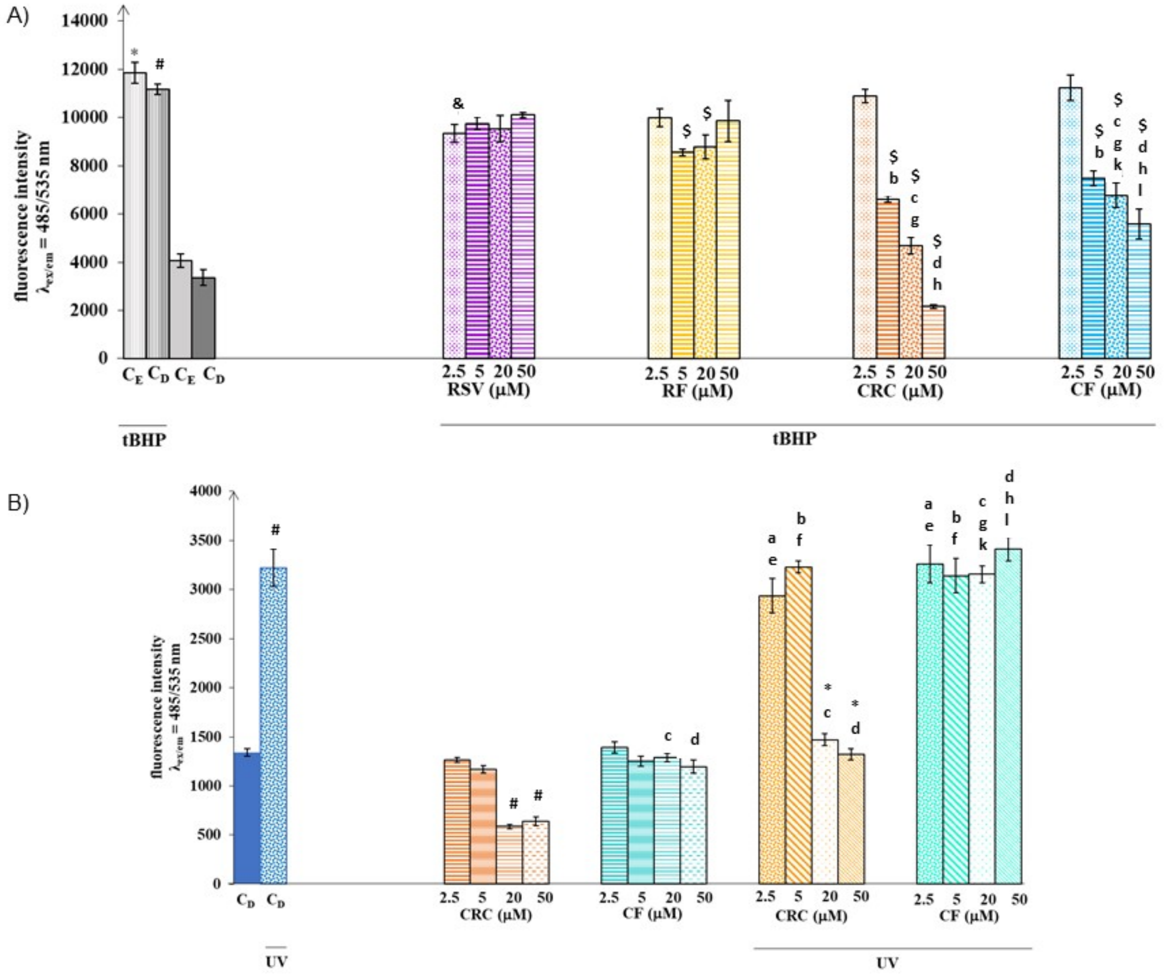

Figure S2

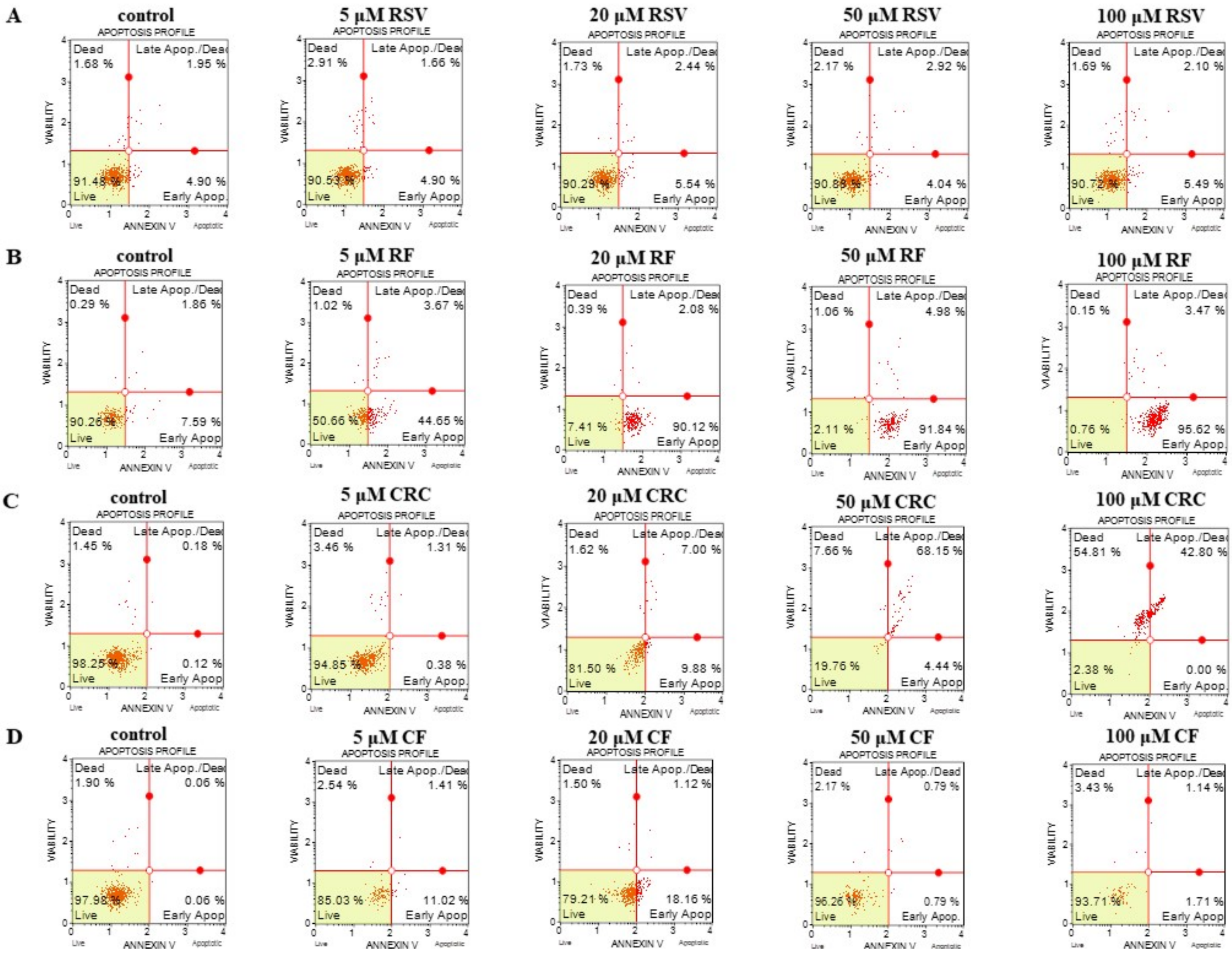
A
control
5 μM RSV
20 μM RSV
50 μM RSV
100 μM RSV
APOPTOSIS PROFILE
Dead
Late Apop./Dead
Live
Early Apop.
VIABILITY
ANNEXIN V
Live
Apoptotic
1.68 % 1.95 % 91.48 % 4.90 %
2.91 % 1.66 % 90.53 % 4.90 %
1.73 % 2.44 % 90.29 % 5.54 %
2.17 % 2.92 % 90.88 % 4.04 %
1.69 % 2.10 % 90.72 % 5.49 %
B
control
5 μM RF
20 μM RF
50 μM RF
100 μM RF
0.29 % 1.86 % 90.26 % 7.59 %
1.02 % 3.67 % 50.66 % 44.65 %
0.39 % 2.08 % 7.41 % 90.12 %
1.06 % 4.98 % 2.11 % 91.84 %
0.15 % 3.47 % 0.76 % 95.62 %
C
control
5 μM CRC
20 μM CRC
50 μM CRC
100 μM CRC
1.45 % 0.18 % 98.25 % 0.12 %
3.46 % 1.31 % 94.85 % 0.38 %
1.62 % 7.00 % 81.50 % 9.88 %
7.66 % 68.15 % 19.76 % 4.44 %
54.81 % 42.80 % 2.38 % 0.00 %
D
control
5 μM CF
20 μM CF
50 μM CF
100 μM CF
1.90 % 0.06 % 97.98 % 0.06 %
2.54 % 1.41 % 85.03 % 11.02 %
1.50 % 1.12 % 79.21 % 18.16 %
2.17 % 0.79 % 96.26 % 0.79 %
3.43 % 1.14 % 93.71 % 1.71 %

Figure S3

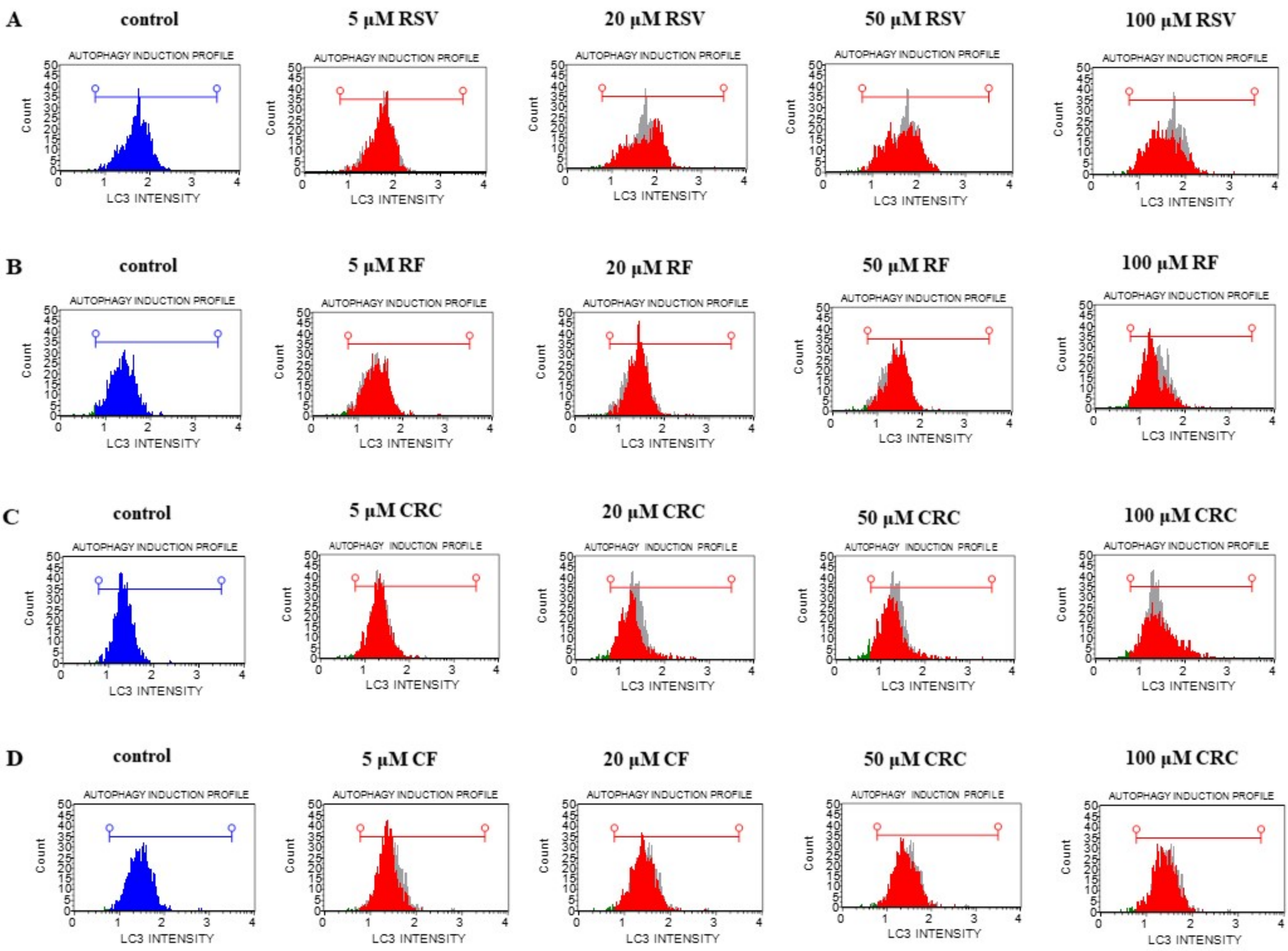

Figure S4

Disclaimer/Publisher's Note: The statements, opinions and data contained in all publications are solely those of the individual author(s) and contributor(s) and not of MDPI and/or the editor(s). MDPI and/or the editor(s) disclaim responsibility for any injury to people or property resulting from any ideas, methods, instructions or products referred to in the content.
